# An analysis of perinatal factors of low T3 syndrome in preterm neonates with a gestational age of 28–35 weeks

**DOI:** 10.1080/07853890.2021.1985731

**Published:** 2021-10-01

**Authors:** Xin Lin, Xian Chen, Chang-yi Yang

**Affiliations:** a Department of Neonatal, Fujian Maternity and Child Health Hospital, Affiliated Hospital of Fujian Medical University, Fuzhou, China; b Department of Obstetrics, Fujian Maternity and Child Health Hospital, Affiliated Hospital of Fujian Medical University, Fuzhou, China

**Keywords:** Low T3 syndrome, analysis, perinatal factor, preterm neonates, 28–35 weeks

## Abstract

**Objective:**

Low triiodothyronine syndrome (LT3S) is a common endocrine disease in preterm neonates. Various serious acute or chronic diseases result in LT3S. Few studies have investigated the causal relationship between perinatal factors and LT3S in preterm neonates with a gestational age (GA) of 28–35 weeks. The present study comprehensively analyzed the perinatal factors of LT3S in preterm neonates.

**Methods:**

This was a retrospective study of neonates with and without LT3S from January 2018 to November 2019. Compared to 206 preterm neonates without LT3S, 158 neonates were diagnosed with LT3S, excluding neonates with congenital malformations, other endocrine diseases, genetic diseases and inherited metabolic diseases.

**Results:**

Five perinatal risk factors for LT3S were confirmed using univariate and multivariate analyses: smaller gestational age, lower birth weight, respiratory distress syndrome (RDS), neonatal sepsis, and dopamine use.

**Conclusions:**

LT3S in preterm neonates was associated with multiple perinatal factors, including smaller gestational age, lower birth weight, RDS, sepsis, and dopamine use. Preterm neonates with a GA of 28–35 weeks who are exposed to a series of high-risk perinatal factors must be closely observed, diagnosed early and treated for primary diseases promptly to reduce the occurrence of LT3S and improve the outcomes.

**Key Message:**
1. Few studies have investigated the relationship between perinatal factors and Low triiodothyronine syndrome (LT3S) in preterm neonates with a gestational age (GA) of 28-35 weeks.
2. LT3S was associated with multiple perinatal factors, including smaller gestational age, lower birth weight, respiratory distress syndrome (RDS), sepsis, and dopamine use.

## Introduction

Low triiodothyronine syndrome (LT3S) is a common endocrine disease in preterm neonates. Reichlin [1] first discovered LT3S in the serum of patients with advanced non-thyroid diseases. Various serious acute and chronic diseases result in LT3S [2,3]. It is become increasingly important to trace the perinatal factors of LT3S in preterm neonates. Because placental monocarboxylic acid transporter eight (MCT8) mRNA is significantly increased in intrauterine growth restriction (IUGR), and neonates with IUGR have foetal hypothyroidism and varying degrees of decreased FT3 and FT4 during the catch-up period of child growth and development [4,5]. Kobayashi found that children with severe asphyxia at birth had transient low thyroid hormone levels 24–48 h after birth. Serum FT3 and FT4 between 72 and 96 h after birth predicted the degree of asphyxia-induced brain damage in neonates [6]. A prospective experimental study confirmed that preterm neonates with respiratory distress syndrome (RDS) showed decreased T3 values on the fifth day after birth but no significant change in thyroid-stimulating hormone (TSH) values [3]. The relationship between perinatal factors, such as neonatal sepsis and prenatal dexamethasone (DXM) use, and LT3S in preterm neonates was discussed in some articles [7,8]. However, whether these risk factors result in LT3S in preterm neonates with a gestational age (GA) of 28–35 weeks requires further comprehensive retrospective study.

Some studies have reported that a lack of sufficient thyroxine in preterm neonates could result in several adverse neonatal outcomes, including dyspnoea [9], feeding intolerance, hypocalcaemia, anaemia, poor neurodevelopment and cerebral palsy [10,11]. Therefore, a comprehensive investigation of the relationship between perinatal factors and preterm infants with and without LT3 syndrome was necessary.

## Materials and methods

### Subjects

A total of 364 preterm neonates at a GA of 28–35 weeks who were born in the Neonatal Department of Fujian Maternal and Child Health Hospital were included. The inclusion criteria were a GA less than 36 weeks and a diagnosis of LT3S between January 2018 and November 2019. All of the neonates in the LT3S group received levothyroxine treatment. Physiological doses of dexamethasone were used in some preterm neonates a few days before the start of labour to promote lung maturity and avoid the occurrence of RDS. Low T3 syndrome in preterm neonates was diagnosed as low T3 levels, low or normal T4 levels and normal TSH [12]. The GA of preterm neonates in the study was between 28 and 35 weeks. The exclusion criteria were preterm neonates with congenital hypothyroidism (who has high TSH, low free T4 or T3), congenital malformations, other endocrine diseases, genetic diseases and inherited metabolic diseases. All mothers were checked during pregnancy and delivered at our hospital, and mothers with incomplete pregnancy data were excluded. Mothers with Grave’s disease and chronic thyroiditis were excluded in the study as well. For each neonate with LT3S, we selected 1 or 2 neonates without LT3S who were born at our hospital simultaneously and were matched in some demographic and neonatal characteristics (control group). Neonates in both groups were examined for thyroid function for the first time 5th–7th days after birth.

### Methods

The tested items were free triiodothyronine (FT3), free thyroxine (FT4), and TSH, and complete follow-up data were collected. FT3, FT4 and TSH levels were measured in peripheral venous blood samples (1–2 ml) using a chemiluminescence immunoassay (CLIA) (Siemens ADVIA Centaur XP) according to the manufacturer’s protocol. LT3S in preterm neonates with a GA of 28–35 weeks was diagnosed according to an FT3 level below 2.30 pg/ml, an FT4 level between 0.89 ng/dl and 1.76 ng/dl, and a TSH level between 0.350 µIU/ml and 4.940 µIU/ml.

We collected data on maternal and neonatal factors. (I) Mother's characteristics were including maternal age, parity, occurrence of premature rupture of membrane, turbid amniotic fluid, and placental abruption and postpartum haemorrhage. Gestational diabetes mellitus (GDM), hypertension in pregnancy, anaemia in pregnancy, and thyroid diseases in pregnancy were collected as maternal factors. (II) Neonatal characteristics: neonate sex and the occurrence of small for GA and foetal distress were collected. Pneumonia, GA, birth weight, RDS, and sepsis as factors of the neonatal neonates, and RDS, pneumonia and sepsis were diagnosed in the first week. The use of dopamine before the first examination of thyroid function and the use of dextromethorphan (DXM) before delivery were considered drug factors. Mothers who are at risk of preterm birth before GA of 35 weeks were given dexamethasone sodium phosphate injection at a dose of 5 mg/12h, and the usage was intramuscular injection with a course of 4 times in total.

### Statistical analyses

Statistical analyses were performed using SPSS Statistic version 26.0 (IBM Corp, Armonk, NY). Continuous data were analyzed using *t*-tests, and categorical variables were analyzed using the *x*^2^ test or binary logistic regression analysis. Chi-squared tests were used to determine the association between LT3S in preterm neonates with a GA of 28–35 weeks and possible perinatal risk factors in univariate analysis. Fisher’s exact test was used when the variable was only found in a small number of preterm neonates (*n* < 5). Binary logistic regression analysis was performed to estimate the relationship between LT3S in preterm neonates with a GA of 28–35 weeks and perinatal risk factors found on univariate factor analysis. Odds ratios (ORs) were used to measure the risk factors for LT3S in preterm neonates with a GA of 28–35 weeks, and bivariate correlation coefficients were used to estimate whether there was a positive or negative relationship. Results with *p* values <.05 were considered statistically significant.

## Results

We included 364 preterm neonates with a GA of 28–35 weeks, including 158 neonates with LT3S (LT3S group) and 206 neonates without LT3S (NO-LT3S group). The demographic characteristics of the neonates and mothers in both groups were not significantly different (*p* > .05), as shown in Table 1. The mean GA was 30.8 ± 1.8 weeks in the LT3S group, the mean birth weight was 1451 ± 282.5 g, and the mean FT3 value was 1.89 ng/dl. The mean GA was 33.3 ± 1.6 weeks in the NO-LT3S group, the mean birth weight was 1977 ± 401.8 g, and the mean FT3 value was 2.72 ng/dl. Most cases of LT3S occurred in neonates with a GA between 28 and 32 weeks (72.15%) and a birth weight of <1500 g (63.29%).

**Table 1.** Relationship between perinatal factors and low T3 syndrome in prematurity (28–35weeks).

Variables	LT3S	NO-LT3S	*p* Value
	(*n* = 158)	(*n* = 206)	
Mother's characteristics			
Mothers' age			
≤ 35 years	119	154	.903*
> 35 years	39	52	
Multiple pregnancy (Y/N)	57/101	61/145	.192
Premature rupture of membranes (Y/N)	55/103	81/125	.378
Turbid amniotic fluid (Y/N)	32/126	35/171	.426
Placental abruption (Y/N)	24/134	25/181	.397
Postpartum haemorrhage (Y/N)	5/153	4/202	.686
Neonatal characteristics			
Male/female	86/72	111/95	.917*
Small for gestational age (Y/N)	31/127	26/180	.069
Foetal distress (Y/N)	34/124	39/167	.541
Mother's factors			
GDM (Y/N)	37/121	49/157	.935
Hypertension in pregnancy (Y/N)	20/138	39/167	.107
Anaemia in pregnancy (Y/N)	66/92	77/129	.395
Thyroid diseases in pregnancy (Y/N)	13/145	24/182	.284
Neonatal factors			
Gestational age			
28–32 weeks	114	35	<.01
32–33 weeks	32	83	
34–35 weeks	12	88	
Birth weight			
<1500 g	100	19	<.01
1500–2499 g	57	167	
≥ 2500 g	1	20	
RDS (Y/N)	43/115	18/188	<.01
Pneumonia (Y/N)	146/12	162/44	<.01
Sepsis (Y/N)	36/122	15/191	<.01
DXM (Y/N)	154/4	180/26	<.01
Dopamine (Y/N)	52/106	24/182	<.01

GDM: gestational diabetes mellitus; RDS: respiratory distress syndrome; DXM: dexamethasone; Y: YES; N: NO.

*Overall *x*²test.

Chi-squared tests showed that only seven perinatal factors were significantly different (*p* < .05). Analyses of maternal factors revealed that GDM, hypertension in pregnancy, anaemia in pregnancy, and thyroid diseases were not related with LT3S in neonates (*p* > .05). Neonatal factors, including smaller gestational age, lower birth weight, RDS, sepsis, and pneumonia, showed significant differences (*p* < .05). Preterm neonates with a GA of 28–35 weeks exposed to DXM and dopamine had a high probability of LT3S (Table 1).

We combined multiple perinatal factors and performed a collinearity analysis (VIF < 3) to determine definite independent risk factors. ORs were used to measure the positive or negative correlations among risk factors and the occurrence of LT3S in preterm neonates with a GA of 28–35 weeks. Binary logistic regression analyses revealed a positive relationship between LT3S in preterm neonates with a GA of 28–35 weeks and perinatal factors, such as smaller gestational age, lower birth weight, RDS, sepsis, and dopamine use (Table 2). The use of DXM before delivery and pneumonia was not different in the binary logistic regression analyses (*p* > .05). Comparison of the ORs of the perinatal factors revealed that the OR of sepsis-induced LT3S was the highest (OR = 2.50, *p* = .04), and the OR for lower birth weight-induced LT3S was the lowest (OR = 0.03, *p* < .01). The explanatory power of the model was 81.9% (Table 2).

**Table 2.** Binary logistic regression models investigating the relationship between exposure to multiple perinatal factors and low T3 syndrome in premature neonates.

						95% CI for OR	
Variables	*B*	S. E	Wald*x*²	*p* Value	OR	Lower	Upper
RDS	0.902	0.41	4.80	.029	2.46	1.10	5.52
Pneumonia	−0.197	0.44	0.20	.656	0.82	0.35	1.96
Sepsis	0.918	0.45	4.23	.040	2.50	1.04	6.00
Dopamine	0.894	0.37	5.85	.016	2.44	1.19	5.04
DXM	0.108	0.71	0.02	.880	1.11	0.28	4.48
Gestational age	−0.286	0.11	6.81	.009	0.75	0.61	0.93
Birth weight	−3.578	0.65	30.78	<.01	0.03	0.01	0.10
Constant	14.516	3.37	18.59	.000			

RDS: respiratory distress syndrome; DXM: dexamethasone.

## Discussion

T3 supports central nervous system development by regulating gene expression, neuron migration and differentiation, axon growth, dendrite development and synapse formation during the formation of cerebral nerves [13]. Inadequate thyroid hormone secretion is closely related to growth defects, poor neurological outcomes in preterm neonates [14], and cerebral palsy in adults [16]. Previous studies examined perinatal factors for congenital hypothyroidism [15] or only a few risk factors for LT3S in preterm neonates [7,8]. We found a relationship between LT3S in preterm neonates with a GA of 28–35 weeks and multiple perinatal factors, including factors involving the mother, neonatal neonates and drugs.

The immaturity of the thyrotropic axis adds complexity to its interpretation particularly in very preterm neonates with a GA of less than 28 weeks. This phenomenon relates to the initial adaptation to critical illness, which is a decrease body temperature and decrease metabolism. Preterm neonates with GA less than 28 weeks were not included in this study. We did not routinely examine thyroid functions in preterm neonates with a GA of more than 36 weeks in the neonatal department, and preterm neonates with a GA of 36–37 weeks were not included in the study.

Because of the immature hypothalamic–pituitary–thyroid axis of preterm neonates, who exhibit a smaller gestational age and lower birth weight, the extent of TSH increases, and the peak point reached by T4 and T3 decreases accordingly [4]. We also found that neonates with smaller gestational age and lower birth weights had increased incidences of LT3S in preterm neonates with a GA of 28–35 weeks. Preterm neonates who were smaller gestational age and had lower birth weight were more likely to have multiple severe diseases, and changes in the iodine-removing pathway caused decreased serum T3 and T4 levels [2]. We confirmed that low total T3 levels were associated with multiple serious illnesses. Although there were no differences between the mother factors and the incidence of LT3S in preterm neonates, a series of neonatal factors were identified. Neonates with sepsis may have intracellular sugar utilization disorders, and neonates with increased cortisol levels showed reduced 5′ deiodinase activity and a reduced conversion rate of T4 to T3 in peripheral tissues [7]. These preterm neonates were also less likely to have LT3S. We found that pneumonia was not different between the two groups because pneumonia was less likely to increase cortisol levels and reduce the conversion rate of T4 to T3 in peripheral tissues, except severe pneumonia. We did not include severe pneumonia. RDS positively correlated with the incidence of LT3S and resulted in increased serum interleukin-6 (IL-6) and tumour necrosis factor alpha (TNF-α) levels [17,18]. Filippiha found that the use of dopamine in very low birth weight neonates led to a reduction in the levels of TSH, FT3, and FT4 [19]. A mouse model [20] showed that the reduction in thyroid hormone levels affected the central dopaminergic nervous system activity of mice, reduced the D1 dopamine function of the nigrostriatal pathway, inhibited neurotransmitter transmission, and caused the mice to appear indifferent to a new environment. We confirmed the positive risk (OR = 2.44) of dopamine use resulting in LT3S in preterm neonates with a GA of 28–35 weeks, and we suggest that the use of dopamine be reduced in preterm neonates at risk of LT3S. The use of DXM to treat postpartum lung diseases in preterm neonates with a GA less than 28 weeks inhibited the release of TSH and reduced the conversion of T4 to T3 in peripheral tissues. The FT3 level dropped significantly [8], the FT4 level increased [21], and these levels returned to normal once DXM treatment was stopped. In our study, although univariate analysis found a significant difference in the use of DXM before delivery between the two groups, there were no differences in binary logistic regression analysis after correction for confounding factors (*p* = .88) in preterm neonates with a GA of 28–35 weeks.

FT3 promotes the movements of the intestine and functions of the digestive glands. Previous research showed that intestinal peristalsis was reduced with insufficient secretion of FT3, and excessive bacterial growth occurs, which increases the likelihood of NEC [7,22]. FT3 participates in the regulation of substance metabolism and promotes the decomposition of bone matrix proteins [10]. The reduction in FT3 also affected haemoglobin synthesis in the neonatal period [11,22] and may result in anaemia.

One of the advantages of our study is that it involved a large number of neonates, including neonates with a GA of 28–35 weeks over a period of almost 2 years. Another advantage is that this study was a single-centre study, which minimizes differences between the groups. The primary diseases of all preterm neonates diagnosed with LT3S were treated in a timely manner, and the FT3 levels eventually returned to normal.

One limitation of our study is that it was a retrospective study of preterm neonates with a GA of 28–35 weeks over a period of 2 years. Therefore, we will make efforts to perform prospective research to determine definite perinatal factors for LT3S in preterm neonates (GA ≥ 36 weeks or GA < 28 weeks) and full-term neonates in the future. Long-term follow-up and comparisons of neurological outcomes of neonates in the LT3S and NO-LT3S groups throughout childhood are necessary, and we will track these data continuously.

## Conclusions

LT3S in preterm neonates is a complex syndrome associated with multiple perinatal factors, including smaller gestational age, lower birth weight, RDS, sepsis, and dopamine use. Therefore, preterm neonates with a GA of 28–35 weeks exposed to a series of high-risk perinatal factors must be closely observed, diagnosed early and treated for primary diseases promptly to reduce the occurrence of LT3S and improve the outcomes.

## Supplementary Material

Supplemental Material

## Data Availability

Access to data is regulated by Chinese law. Data are available from the Fujian Maternity and Child Health Hospital for researchers who meet the criteria as required by the Chinese law for access to confidential data. The authors confirm that the data supporting the findings of this study are available within the article and its supplementary materials.
